# Supplementary material for: A scoping review of evidence of naturally occurring Japanese encephalitis infection in vertebrate animals other than humans, ardeid birds and pigs
Source: PLoS Negl Trop Dis. 2024 Oct 4;18(10):e0012510. doi: 10.1371/journal.pntd.0012510 (PMC11482687; doi:10.1371/journal.pntd.0012510)
Supplement: S1 Text — Table A Search terms in a scoping review of direct and indirect evidence of naturally occurring Japanese encephalitis virus infection in vertebrate animals other than humans, ardeid birds and pigs. (DOCX) [file pntd.0012510.s001.docx]

**A scoping review of evidence of naturally occurring Japanese encephalitis infection in vertebrate animals other than humans, ardeid birds and pigs**

Zoë A. Levesque^1*^, Michael G. Walsh^2, 3, 4, 5^, Cameron E. Webb^3, 6,7^, Ruth N. Zadoks^1, 3^, Victoria J. Brookes^1, 3^

^1^Sydney School of Veterinary Science, Faculty of Science, The University of Sydney, Camperdown, New South Wales, Australia

^2^Sydney School of Public Health, Faculty of Medicine and Health, The University of Sydney, Camperdown, New South Wales, Australia

^3^Sydney Infectious Diseases Institute, Faculty of Medicine and Health, The University of Sydney, Westmead, New South Wales, Australia

^4^One Health Centre, The Prasanna School of Public Health, Manipal Academy of Higher Education, Manipal, Karnataka, India

^5^The Prasanna School of Public Health, Manipal Academy of Higher Education, Manipal, Karnataka, India

^6^Department of Medical Entomology, NSW Health Pathology, Westmead Hospital, Westmead, New South Wales, Australia

^7^School of Medical Sciences, Faculty of Medicine and Health, The University of Sydney, Camperdown, New South Wales, Australia

*Corresponding author: victoria.brookes@sydney.edu.au

**Supplementary Material**

**Table A** Search terms in a scoping review of direct and indirect evidence of naturally occurring Japanese encephalitis virus infection in vertebrate animals other than humans, ardeid birds and pigs.

| Latest search date | 27 January 2024 |
| --- | --- |
| Web of Science (all databases), <https://www.webofscience.com/> | “Japanese Encephalitis” OR JEV OR JE (Title) and detection OR detected (Topic) |
| Scopus, <https://www.scopus.com/> | “Japanese Encephalitis” OR jev OR je (Article title) and detection OR detected (All fields) |
| ProQuest Central, <https://www.proquest.com/central> | title("Japanese encephalitis" OR "JEV" OR "JE") AND (detection OR detected); Peer reviewed |
| Google Scholar, <https://scholar.google.com.au/> | Exact phrase in title: “Japanese encephalitis”; date range 1935-1980 |


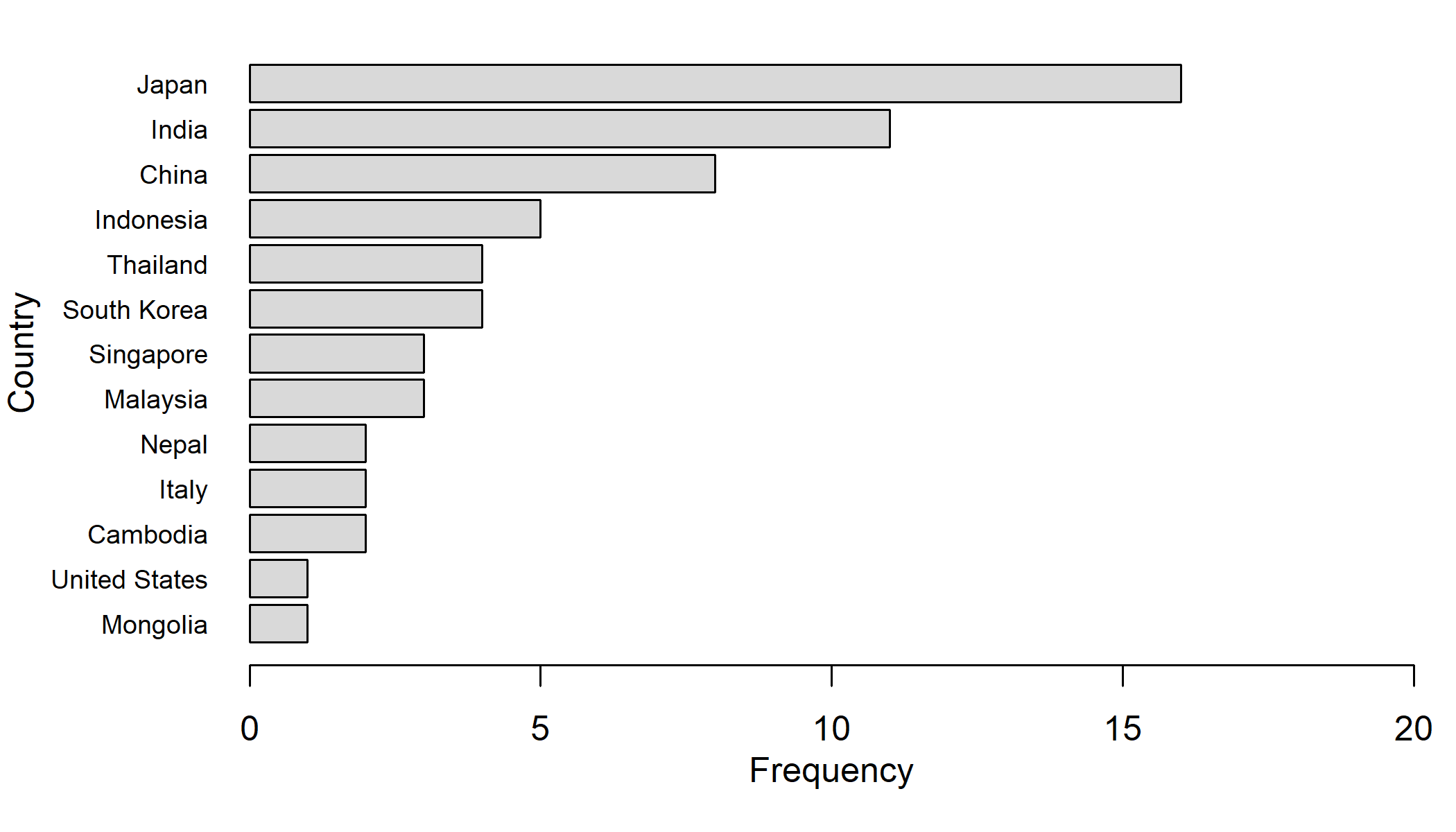

**Fig A** Bar plot of the frequency of study location by country, in a scoping review of direct and indirect evidence of naturally occurring Japanese encephalitis virus infection in vertebrate animals other than humans, ardeid birds and pigs.

**
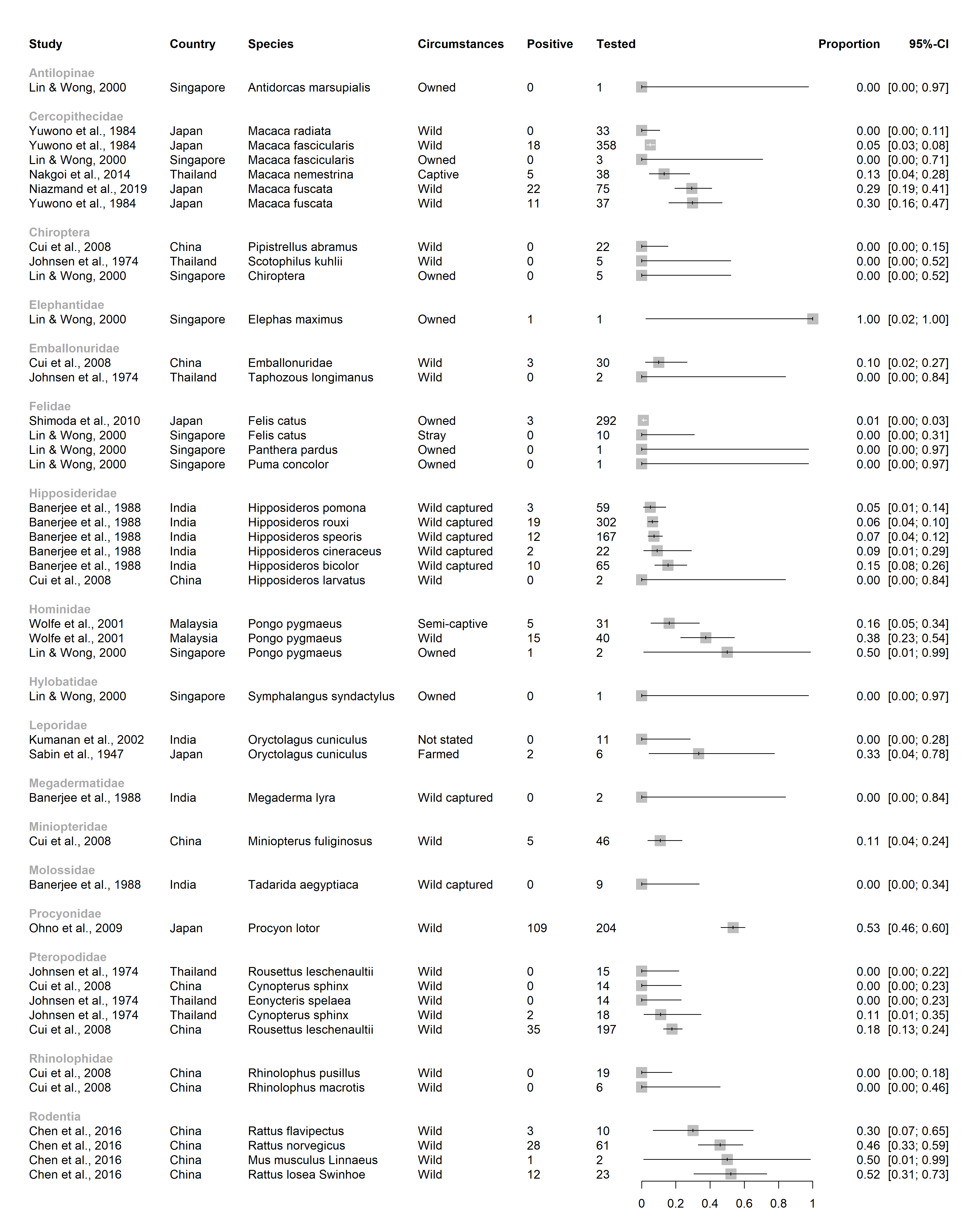
**

**Fig B** Reported seroprevalence (indirect detection of JEV infection [antibody]) in studies of mammals other than Bovinae, Canidae, Caprinae, Equidae, Suidae, humans and ardeid birds. Horizontal lines = 95% confidence intervals.


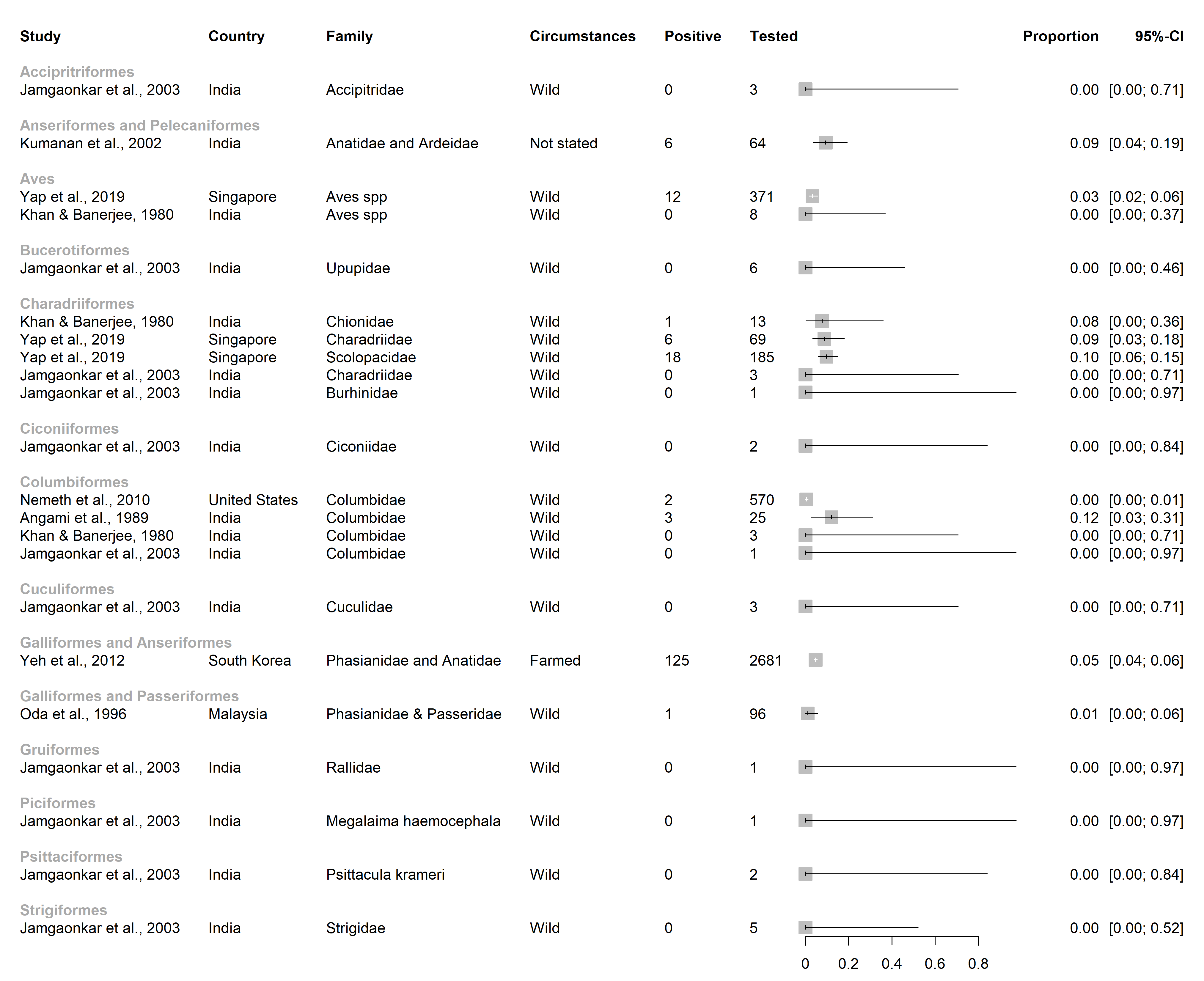


**Fig C** Reported seroprevalence (indirect detection of JEV infection [antibody]) in bird orders in which maximum seroprevalence was <10% in a scoping review of direct and indirect evidence of naturally occurring Japanese encephalitis virus infection in vertebrate animals other than humans, ardeid birds and pigs. Ardeidae (Pelecaniformes) are included from studies in which vertebrate animals other than pigs and humans were also tested. Horizontal lines = 95% confidence intervals.

**
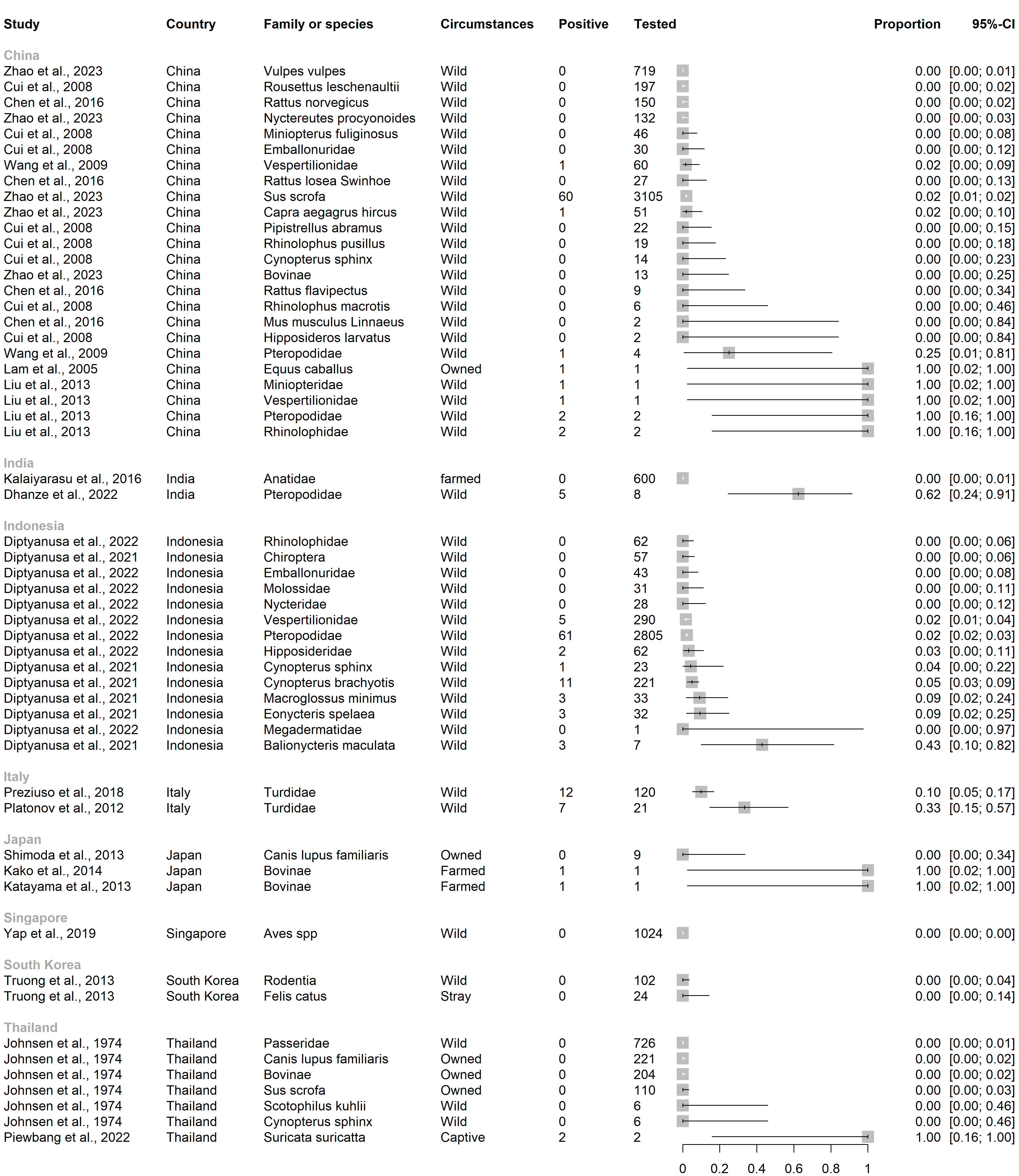
 Fig D** Reported prevalence (direct detection of JEV infection [virus, viral antigen, or viral RNA]) stratified by country in a scoping review of direct and indirect evidence of naturally occurring Japanese encephalitis virus infection in vertebrate animals other than humans, ardeid birds and pigs. Suidae are included from studies in which vertebrate animals other than ardeid birds and humans were also tested. Horizontal lines = 95% confidence intervals.


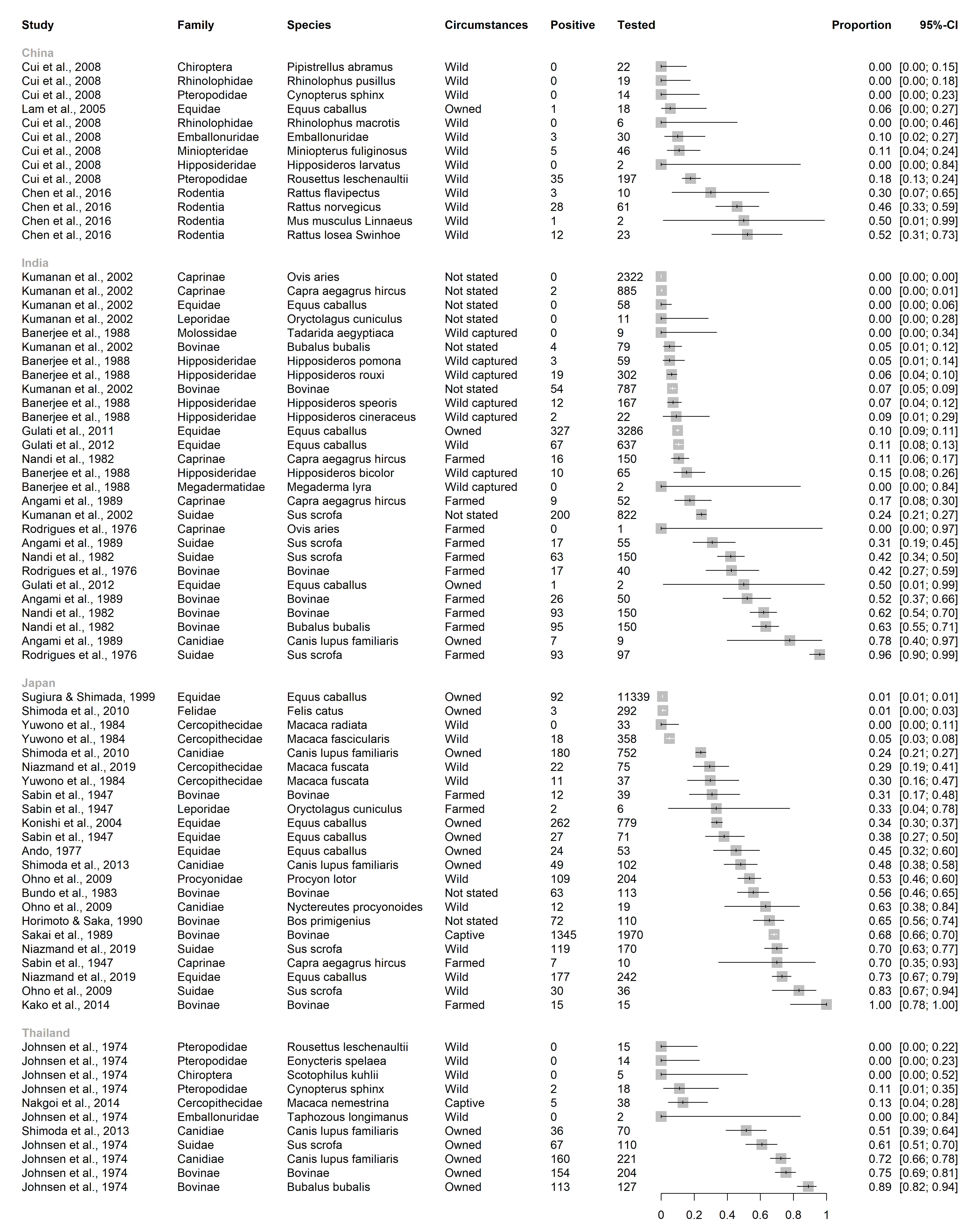


**Fig E** Reported seroprevalence (indirect detection of JEV infection [antibody]) of mammals in China, India, Japan, and Thailand, in a scoping review of direct and indirect evidence of naturally occurring Japanese encephalitis virus infection in vertebrate animals other than humans, ardeid birds and pigs. Suidae are included from studies in which vertebrate animals other than ardeid birds and humans were also tested. Horizontal lines = 95% confidence intervals.


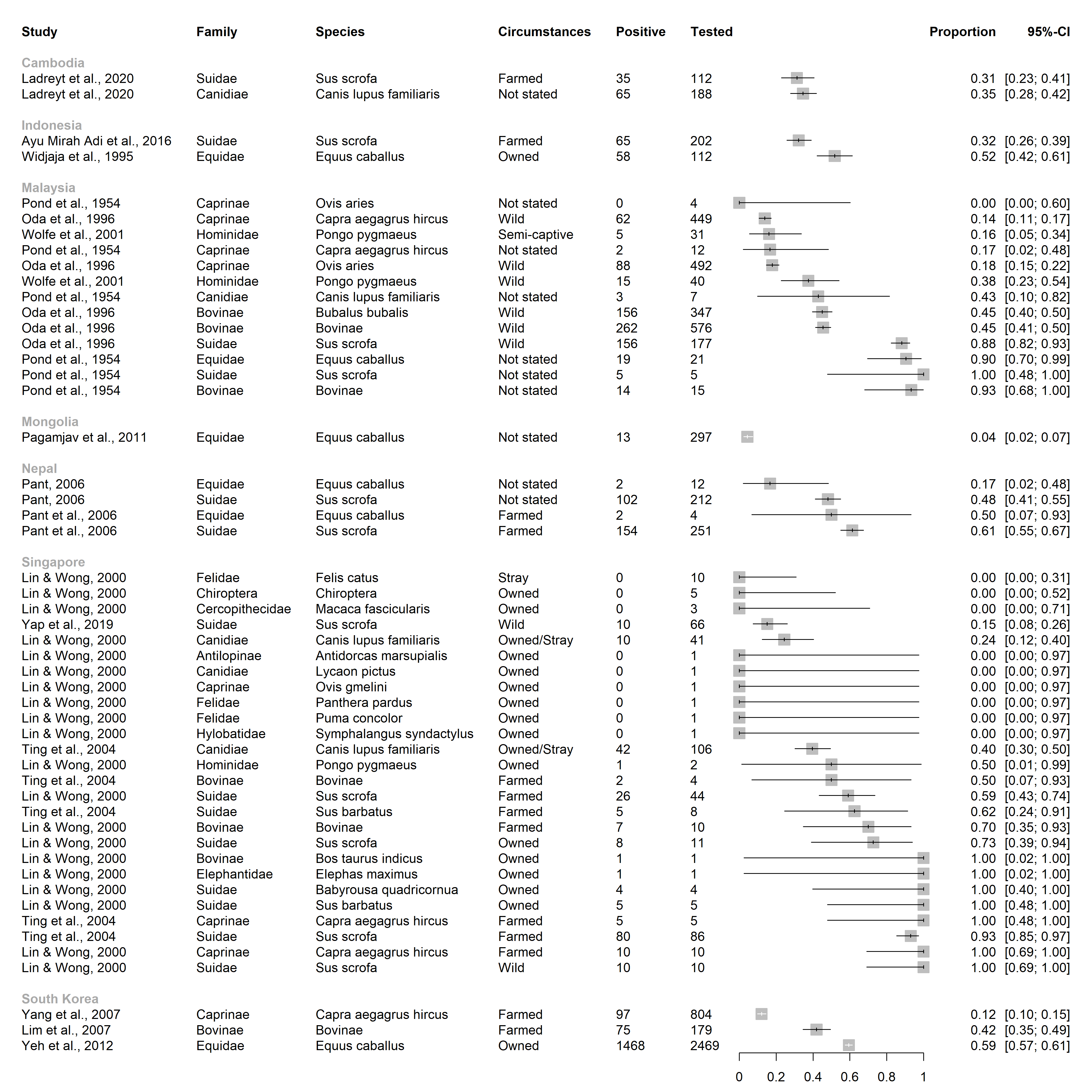


**Fig F** Reported seroprevalence (indirect detection of JEV infection [antibody]) of mammals in Cambodia, Indonesia, Mongolia, Nepal, Singapore and South Korea in a scoping review of direct and indirect evidence of naturally occurring Japanese encephalitis virus infection in vertebrate animals other than humans, ardeid birds and pigs. Suidae are included from studies in which vertebrate animals other than ardeid birds and humans were also tested. Horizontal lines = 95% confidence intervals.


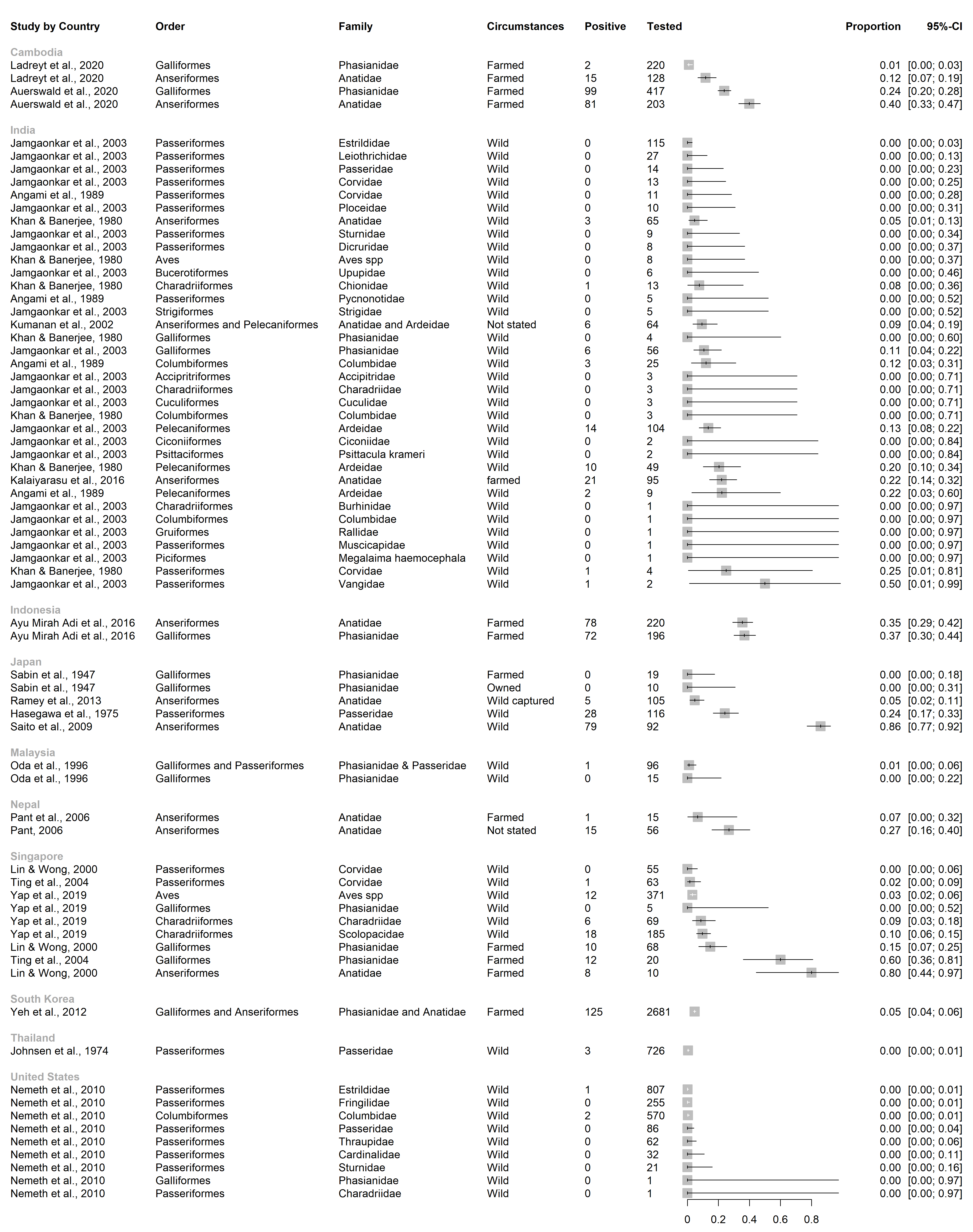


**Fig G** Reported seroprevalence (indirect detection of JEV infection [antibody]) of birds by country in a scoping review of direct and indirect evidence of naturally occurring Japanese encephalitis virus infection in vertebrate animals other than humans, ardeid birds and pigs. Suidae are included from studies in which vertebrate animals other than ardeid birds and humans were also tested. Horizontal lines = 95% confidence intervals.

**References**

1. Lin YN, Wong WK. Sero-prevalence of Japanese encephalitis virus in various species of animals in Singapore - a preliminary study. Singapore Journal of Primary Industries. 2000;28:57-61. PubMed PMID: CABI:20013104959.

2. Yuwono J, Suharyono W, Koiman I, Tsuchiya Y, Tagaya I. Seroepidemiological survey on dengue and Japanese encephalitis virus infections in Asian monkeys. The Southeast Asian Journal of Tropical Medicine and Public Health. 1984;15(2):194-200.

3. Nakgoi K, Nitatpattana N, Wajjwalku W, Pongsopawijit P, Kaewchot S, Yoksan S, et al. Dengue, Japanese encephalitis and Chikungunya virus antibody prevalence among captive monkey (*Macaca nemestrina*) colonies of Northern Thailand. American Journal of Primatology. 2014;76(1):97-102. doi: 10.1002/ajp.22213.

4. Niazmand MH, Hirai T, Ito S, Habibi WA, Noori J, Hasheme R, et al. Causes of death and detection of antibodies against Japanese encephalitis virus in Misaki feral horses (*Equus caballus*) in Southern Japan, 2015–17. Journal of Wildlife Diseases. 2019;55(4):804-11. doi: 10.7589/2018-10-265.

5. Cui J, Counor D, Shen D, Sun G, He H, Deubel V, et al. Detection of Japanese encephalitis virus antibodies in bats in southern China. American Journal of Tropical Medicine and Hygiene. 2008;78(6):1007-11. doi: 10.4269/ajtmh.2008.78.1007. PubMed PMID: WOS:000256504500030.

6. Johnsen DO, Edelman R, Grossman RA, Muangman D, Pomsdhit J, Gould DJ. Study of Japanese encephalitis virus in Chiangmai Valley, Thailand V. Animal infections. American Journal of Epidemiology. 1974;100(1):57-68. doi: 10.1093/oxfordjournals.aje.a112009.

7. Shimoda HO, Yoshito; Mochizuki, Masami; Iwata, Hiroyuki; Okuda, Masaru; Maeda, Ken. Dogs as Sentinels for Human Infection with Japanese Encephalitis Virus. Emerging Infectious Diseases. 2010;16(7):1137-9. doi: 10.3201/eid1607.091757. PubMed PMID: WOS:000279522200016.

8. Banerjee K, Bhat HR, Geevarghese G, Jacob PG, Malunjkar AS. Antibodies against Japanese encephalitis virus in insectivorous bats from Karnataka. Indian Journal of Medical Research. 1988;87:527-30. PubMed PMID: WOS:A1988N935800001.

9. Wolfe ND, Kilbourn AM, Karesh WB, Rahman HA, Bosi EJ, Cropp BC, et al. Sylvatic transmission of arboviruses among Bornean orangutans. American Journal of Tropical Medicine and Hygiene. 2001;64(5-6):310-6. doi: 10.4269/ajtmh.2001.64.310. PubMed PMID: WOS:000169921600017.

10. Kumanan K, Ramesh A, Velmurugan R, Jagannathan S, Padmanaban VD. Seroepidemiology of Japanese encephalitis among animals and birds in Tamil Nadu. Indian Veterinary Journal. 2002;79(4):311-5. PubMed PMID: WOS:000174895800001.

11. Ohno Y, Sato H, Suzuki K, Yokoyama M, Uni S, Shibasaki T, et al. Detection of antibodies against Japanese encephalitis virus in raccoons, raccoon dogs and wild boars in Japan. Journal of Veterinary Medical Science. 2009;71(8):1035-9. doi: 10.1292/jvms.71.1035.

12. Chen S-w, Jiang L-n, Zhong X-s, Zheng X-y, Ma S-j, Xiong Y-q, et al. Serological Prevalence Against Japanese Encephalitis Virus-Serocomplex Flaviviruses in Commensal and Field Rodents in South China. Vector-Borne and Zoonotic Diseases. 2016;16(12):777-80. doi: 10.1089/vbz.2015.1934. PubMed PMID: WOS:000390243200007.

13. Jamgaonkar AV, Yergolkar PN, Geevarghese G, Joshi GD, Joshi MV, Mishra AC. Serological evidence for Japanese encephalitis virus and West Nile virus infections in water frequenting and terrestrial wild birds in Kolar District, Karnataka State, India. A retrospective study. Acta Virologica. 2003;47(3):185-8.

14. Yap G, Lim XF, Chan S, How CB, Humaidi M, Yeo G, et al. Serological evidence of continued Japanese encephalitis virus transmission in Singapore nearly three decades after end of pig farming. Parasites and Vectors. 2019;12(1). doi: 10.1186/s13071-019-3501-0.

15. Nemeth NM, Bosco-Lauth AM, Sciulli RH, Gose RB, Nagata MT, Bowen RA. Serosurveillance for Japanese encephalitis and West Nile viruses in resident birds in Hawai'i. Journal of Wildlife Diseases. 2010;46(2):659-64. doi: 10.7589/0090-3558-46.2.659.

16. Khan FU, Banerjee K. Mosquito collection in heronries and antibodies to Japanese encephalitis-virus in birds in Asansol-Dhanbad region. Indian Journal of Medical Research. 1980;71(JAN):1-5. PubMed PMID: WOS:A1980JF65600001.

17. Angami K, Chakravarty SK, Das MS, Chakraborty MS, Mukherjee KK. Seroepidemiological study of Japanese encephalitis in Dimapur, Nagaland. Journal of Communicable Diseases. 1989;21(2):87-95.

18. Yeh JY, Lee JH, Park JY, Seo HJ, Moon JS, Cho IS, et al. A diagnostic algorithm to serologically differentiate West Nile virus from Japanese encephalitis virus infections and its validation in field surveillance of poultry and horses. Vector-Borne and Zoonotic Diseases. 2012;12(5):372-9. doi: 10.1089/vbz.2011.0709.

19. Oda K, Igarashi A, Kheong CT, Hong CC, Vijayamalar B, Sinniah M, et al. Cross-sectional serosurvey for Japanese encephalitis specific antibody from animal sera in Malaysia 1993. Southeast Asian Journal of Tropical Medicine and Public Health. 1996;27(3):463-70.

20. Zhao G, Gao Y, Shi N, Zhang S, Xiao P, Zhang J, et al. Molecular Detection and Genetic Characterization of Japanese Encephalitis Virus in Animals from 11 Provinces in China. Viruses. 2023;15(3). doi: 10.3390/v15030625.

21. Wang JL, Pan XL, Zhang HL, Fu SH, Wang HY, Tang Q, et al. Japanese encephalitis viruses from bats in Yunnan, China. Emerging Infectious Diseases. 2009;15(6):939-42. doi: 10.3201/eid1506.081525.

22. Lam KHK, Ellis TM, Williams DT, Lunt RA, Daniels PW, Watkins KL, et al. Japanese encephalitis in a racing thoroughbred gelding in Hong Kong. Veterinary Record. 2005;157(6):168-73. doi: 10.1136/vr.157.6.168.

23. Liu S, Li X, Chen Z, Chen Y, Zhang Q, Liao Y, et al. Comparison of genomic and amino acid sequences of eight Japanese encephalitis virus isolates from bats. Archives of Virology. 2013;158(12):2543-52. doi: 10.1007/s00705-013-1777-5.

24. Kalaiyarasu S, Mishra N, Khetan RK, Singh VP. Serological evidence of widespread West Nile virus and Japanese encephalitis virus infection in native domestic ducks (*Anas platyrhynchos* var *domesticus*) in Kuttanad region, Kerala, India. Comparative Immunology, Microbiology and Infectious Diseases. 2016;48:61-8. doi: 10.1016/j.cimid.2016.08.002.

25. Dhanze H, Karikalan M, Mehta D, Gupta M, Mote A, Kumar M, et al. First report on the detection of Japanese encephalitis virus in fruit bats from India. Journal of Vector Borne Diseases. 2022;59(2):190-2. doi: 10.4103/0972-9062.335769.

26. Diptyanusa A, Herini ES, Indarjulianto S, Satoto TBT. Estimation of Japanese encephalitis virus infection prevalence in mosquitoes and bats through nationwide sentinel surveillance in Indonesia. PLoS One. 2022;17(10):e0275647-e. PubMed PMID: ZOOREC:ZOOR15907050797.

27. Diptyanusa A, Herini ES, Indarjulianto S, Satoto TBT. The detection of Japanese encephalitis virus in Megachiropteran bats in West Kalimantan, Indonesia: A potential enzootic transmission pattern in the absence of pig holdings. International Journal for Parasitology: Parasites and Wildlife. 2021;14:280-6. doi: 10.1016/j.ijppaw.2021.03.009.

28. Preziuso S, Mari S, Mariotti F, Rossi G. Detection of Japanese Encephalitis Virus in bone marrow of healthy young wild birds collected in 1997–2000 in Central Italy. Zoonoses and Public Health. 2018;65(7):798-804. doi: 10.1111/zph.12501.

29. Platonov AE, Rossi G, Karan LS, Mironov KO, Busani L, Rezza G. Does the Japanese encephalitis virus (JEV) represent a threat for human health in Europe? Detection of JEV RNA sequences in birds collected in Italy. Eurosurveillance. 2012;17(32). doi: 10.2807/ese.17.32.20241-en.

30. Kako N, Suzuki S, Sugie N, Kato T, Yanase T, Yamakawa M, et al. Japanese encephalitis in a 114-month-old cow: pathological investigation of the affected cow and genetic characterization of Japanese encephalitis virus isolate. BMC Veterinary Research. 2014;10(1). doi: 10.1186/1746-6148-10-63.

31. Katayama T, Saito S, Horiuchi S, Maruta T, Kato T, Yanase T, et al. Nonsuppurative encephalomyelitis in a calf in Japan and isolation of Japanese encephalitis virus genotype 1 from the affected calf. Journal of Clinical Microbiology. 2013;51(10):3448-53. doi: 10.1128/JCM.00737-13.

32. Piewbang C, Wardhani SW, Chaiyasak S, Yostawonkul J, Kasantikul T, Techangamsuwan S. Japanese encephalitis virus infection in meerkats (*Suricata suricatta*). Zoonoses and Public Health. 2022;69(1):55-60. doi: 10.1111/zph.12882.

33. Truong QL, Seo TW, Yoon BI, Kim HC, Han JH, Hahn TW. Prevalence of swine viral and bacterial pathogens in rodents and stray cats captured around pig farms in Korea. Journal of Veterinary Medical Science. 2013;75(12):1647-50. doi: 10.1292/jvms.12-0568.

34. Gulati BR, Singha H, Singh BK, Virmani N, Khurana SK, Singh RK. Serosurveillance for Japanese encephalitis virus infection among equines in India. Journal of Veterinary Science. 2011;12(4):341-5. doi: 10.4142/jvs.2011.12.4.341.

35. Gulati BR, Singha H, Singh BK, Virmani N, Kumar S, Singh RK. Isolation and genetic characterization of Japanese encephalitis virus from equines in India. Journal of Veterinary Science. 2012;13(2):111-8. doi: 10.4142/jvs.2012.13.2.111.

36. Nandi AK, Mukherjee KK, Chakravarti SK, Chakraborty MS. Activity of Japanese encephalitis virus among certain domestic animals in West Bengal. Indian Journal of Medical Research. 1982;76(OCT):499-503. PubMed PMID: WOS:A1982PM00900001.

37. Rodrigues FM, Vidyasagar J, Bright Singh P, Ghosh SN, Guttikar SN, Joshi MV, et al. The 1973 epidemic of Japanese encephalitis in West Bengal: a serological survey of domestic animals. Indian Journal of Medical Research. 1976;64(7):973-80.

38. Sugiura T, Shimada K. Seroepizootiological Survey of Japanese Encephalitis Virus and Getah Virus in Regional Horse Race Tracks from 1991 to 1997 in Japan. Journal of Veterinary Medical Science. 1999;61(8):877-81. doi: 10.1292/jvms.61.877.

39. Sabin AB, Ginder DR, Matumoto M. Difference in dissemination of the virus of Japanese-B-encephalitis among domestic animals and human beings in Japan. American Journal of Hygiene. 1947;46(3):341-55. doi: 10.1093/oxfordjournals.aje.a119173. PubMed PMID: WOS:A1947YA28500004.

40. Konishi E, Shoda M, Kondo T. Prevalence of antibody to Japanese encephalitis virus nonstructural 1 protein among racehorses in Japan: Indication of natural infection and need for continuous vaccination. Vaccine. 2004;22(9-10):1097-103. doi: 10.1016/j.vaccine.2003.10.001.

41. Ando Y. Serological epizootiology of Japanese encephalitis virus infection in horses in Japan. Experimental Reports of Equine Health Laboratory. 1977;(14):38-53. PubMed PMID: WOS:A1977EC35600005.

42. Bundo K, Morita K, Igarashi A. Antibodies against Japanese encephalitis virus in bovine sera in Nagasaki, 1981. Tropical Medicine. 1983;25(2):73-82.

43. Horimoto M, Sakai T. Maternally derived antibodies to Japanese encephalitis virus in cattle. The Journal of the Japanese Association for Infectious Diseases. 1990;64(9):1205-8. doi: 10.11150/kansenshogakuzasshi1970.64.1205.

44. Auerswald H, Ruget AS, Ladreyt H, In S, Mao S, Sorn S, et al. Serological Evidence for Japanese Encephalitis and West Nile Virus Infections in Domestic Birds in Cambodia. Frontiers in Veterinary Science. 2020;7. doi: 10.3389/fvets.2020.00015.

45. Adi AAAM, Astawa NM, Damayanti PAA, Kardena IM, Erawan IGMK, Suardana IW, et al. Seroepidemiological Evidence for the Presence of Japanese Encephalitis Virus Infection in Ducks, Chickens, and Pigs, Bali-Indonesia. Bali Medical Journal. 2016;5(3):189-94. doi: 10.15562/bmj.v5i3.343. PubMed PMID: WOS:000390892900034.

46. Widjaja S, Soekotjo W, Hartati S, Jennings GB, Corwin AL. Prevalence of hemagglutination-inhibition and neutralizing antibodies to arboviruses in horses of Java. Southeast Asian Journal of Tropical Medicine and Public Health. 1995;26(1):109-13. PubMed PMID: BIOSIS:PREV199598555488.

47. Pond WL, Russ SB, Lancaster WE, Audy JR, Smadel JE. Japanese encephalitis in Malaya 2. Distribution of neutralizing antibodies in man and animals. American Journal of Hygiene. 1954;59(1):17-25. doi: 10.1093/oxfordjournals.aje.a119619. PubMed PMID: WOS:A1954XU80300002.

48. Pagamjav O, Kobayashi K, Murakami H, Tabata Y, Miura Y, Boldbaatar B, et al. Serological survey of equine viral diseases in Mongolia. Microbiology and Immunology. 2011;55(4):289-92. doi: 10.1111/j.1348-0421.2011.00312.x.

49. Pant GR, editor A serological survey of pigs, horses, and ducks in Nepal for evidence of infection with Japanese encephalitis virus. Annals of the New York Academy of Sciences; 2006.

50. Pant GR, Lunt RA, Rootes CL, Daniels PW. Serological evidence for Japanese encephalitis and West Nile viruses in domestic animals of Nepal. Comparative Immunology Microbiology and Infectious Diseases. 2006;29(2-3):166-75. doi: 10.1016/j.cimid.2006.03.003. PubMed PMID: WOS:000237987700008.

51. Ting SHL, Tan HC, Wong WK, Ng ML, Chan SH, Ooi EE. Seroepidemiology of neutralizing antibodies to Japanese encephalitis virus in Singapore: continued transmission despite abolishment of pig farming? Acta Tropica. 2004;92(3):187-91. doi: 10.1016/j.actatropica.2004.04.010. PubMed PMID: WOS:000225319600004.

52. Yang D-K, Kweon C-H, Kim B-H, Hwang I-J, Kang M-I, So B-J, et al. The seroprevalence of Japanese encephalitis virus in goats raised in Korea. Journal of Veterinary Science. 2007;8(2):197-9. doi: 10.4142/jvs.2007.8.2.197. PubMed PMID: WOS:000247660100016.

53. Ramey AM, Spackman E, Yeh JY, Fujita G, Konishi K, Uchida K, et al. Antibodies to H5 subtype avian influenza virus and Japanese encephalitis virus in northern pintails (*Anas acuta*) sampled in Japan. Japanese Journal of Veterinary Research. 2013;61(3):117-23.

54. Hasegawa T, Takehara Y, Takahashi K. Natural and experimental infections of Japanese tree sparrows with Japanese encephalitis virus. Archives of Virology. 1975;49(4):373-6. doi: 10.1007/bf01318247. PubMed PMID: WOS:A1975BB72600010.

55. Saito M, Osa Y, Asakawa M. Antibodies to Flaviviruses in Wild Ducks Captured in Hokkaido, Japan: Risk Assessment of Invasive Flaviviruses. Vector-Borne and Zoonotic Diseases. 2009;9(3):253-8. doi: 10.1089/vbz.2008.0111. PubMed PMID: WOS:000267090300004.

56. Sakai T, Horimoto M. Japanese encephalitis virus infection in cattle: Changes in antibody distribution in the central district of Japan during a 4-year period. Preventive Veterinary Medicine. 1989;7(1):39-47. doi: https://doi.org/10.1016/0167-5877(89)90035-4.
